# Nitrous Oxide Gas

**Published:** 1872-06

**Authors:** J. B. Newbrough


### ARTICLE II.

### *Nitrous Oxide Gas.*

#### A Reported Death from its Use.

BY J. B. NEWBROUGH, D.D.S.

On the 20th day of March, 1872, Mrs. O'Shaughnessy, accompanied by a friend, Mrs. Bigley, went to the office of J. B Newbrough, dentist, to have seven or eight loose front teeth extracted. The following is the substance of the testimony of J. B. Newbrough, and it was corroborated by Mrs. Bigley:

"Advised the patient that the extraction in her case would be so easy she need not take gas. She replied, 'It will kill me to have them out without gas. I never could stand it.' I procured a six gallon bag of gas for her and induced her to take the mouth-piece and try it. When she felt the gas coming, she snatched the bag away, and said she would have her teeth out without gas. For three or four minutes we discussed the matter, and I offered to extract her teeth without gas, but at a sight of the forceps she refused again. Then said she would try the gas once more. It was turned on as before, and she got one imperfect breath—as she called it—of gas, and snatched it away again. In the mean time much of the gas had escaped from the bag. After about eight minutes more, during which, Mrs. Bigley, the datient, and myself conversed about the different friends who had taken gas successfully, I then procured a fresh bagful of gas, and again offered it to her. On this occasion she took two inhalations, and again snatched the bag away, saying, 'That stuff won't have any effect on me. Take it away, I'm afraid it will kill me.' She then wiped her mouth with her hankerchief, being entirely wide-awake, and braced herself to have them out without gas, and I extracted them. Immediately thereafter she fainted, her head dropping over sideways. I raised her up and tried to have her spit out the blood, but she had lost all power. To the jury: I at once ordered the lowering of the temperature of the room to sixty degrees; complexion healthy, but becoming rapidly livid, and finally purple. Kept her upright, so as to determine the blood downward. Respiration about fifteen a minute. Sent for Dr. Otis, physician, who arrived in ten minutes after she fainted. Respiration fell rapidly; death ensued in two or three minutes after the physician's arrival. Applied the galvanic battery to the palms of her hands within one minute after she first fainted; produced no effect; assisted her respiration by raising and lowering her chest. Failed from the very first to induce any muscular contraction; she was entirely relaxed all the time. After death, she became instantly blanched in the face.

My nitrous oxide is made from Powers and Weightman's nitrate of ammonia. I am capable of analyzing these chem icals. Nitrous oxide is not a local irritant. Asphyxia can not be produced by nitrous oxide gas suddenly applied. It is an antidote for asphyxia from chloroform. My apparatus is so guaged that the gas is made at a temperature not exceeding 440°.

"Dr. Fessenden N. Otis testified that he was summoned by Mr. Newbrough, and described the condition of the dying woman when he arrived; adopted the usual restoratives, and continued them forty-five minutes, when death ensued. The witness also gave the result of the *post-mortem* examination; there was no disease of the heart; the brain was perfectly exsanguinated in every part; there was no fluid in any of the ventricles; one lung was more engorged than the other, but healthy; formed no opinion f the cause of death."

"Dr. Charles K. Britton also testified as to the result of the *post-mortem* examination, and proved the healthy condition of the body except that one lung was congested; the brain was in a normal state; judged from the condition of the lungs that death was from asphyxia, but could not express an opinion as to the cause of it.

"Dr. Alexander Buchanan, of No. 335 West Thirtieth street, testified that he was present at the *post-mortem* examination, and observed that the lungs were congested; the right lung was bound down by old pleuritic adhesions; the congestion was *post-mortem*; in opinion of witness death was caused by the nervous shock of having her teeth extracted, though the inhalation of nitrous oxide gas might act as a predisposing cause; the upper lobes of the right lung presented appearances of pneumonia; the nitrous oxide gas is not stimulating, but sedative, and witness would not hesitate to administer the gas to persons suffering from cardiac disease, though it would require greater care under the circumstances; if the operation had been discontinued when the deceased expressed a fear that the inhalation of the gas would kill her, death would not have taken place.

"Dr. Endermann, examining chemist of the Board of Health, who had been instructed by the coroner to inspect the apparatus of Dr. Newbrough, testified that in the preparation of the gas, several wash-bottles should be used for its purification; but one bottle was used by Newbrough, and the water that was in it was raised by the process of manufacture to a boiling heat, and containing no atmospheric air, was, therefore, useless as an absorbent for the removal of impurities; the apparatus was unfitted for the preparation of pure gas; after the death of Mrs. O'Shaughnessy, Dr. Newbrough allowed all the gas in the reservoir to escape, and refilled it with some freshly made, so that witness had not an opportunity to test the quality of the former, it is just as easy to prepare chemically pure gas as impure gas; even though Mrs. O'Shaughnessy had spoken during the administration of the gas, if the mouthpiece was not entirely withdrawn, she would continue to inhale it, and in this way be placed under its influence. The nitrate of ammonia was not entirely pure, but was as good as is generally found; the apparatus is capable of making pure gas, provided the gas is not administered within three hours after made; Newbrough informed him that the gas administered to deceased was made two days previous; did not examine the gas which she had inhaled, as the gasometer was empty, but the gas witness made with the apparatus was pure; the bag used by Mr. Newbrough holds about four gallons, with a mouth-piece which the patient puts in the mouth and inhales directly out of the bag, causing the carbonic acid of exhalation to enter the bag; thought that all the mouth-pieces ought to have a safety-valve; thought that deceased inhaled the nitrous oxide gas partly; had some experience of the physical effect of nitrous oxide gas; the face becomes gradually livid, which increases until the patient is under the influence of the gas about fifty seconds after first inhaling it; pupils dilated; if the pulse is weak at first, it generally becomes more rapid and hard, and when the laryngeal stertor begins, the gas is usually removed; the patient comes out from the influence of the gas so as to be conscious in about two or three minutes, but the dizziness continues from about five to ten minutes; the only experience the witness had had of the bad effect of inhaling the gas was the case of a young man who was his assistant, and who took the gas to have a tooth extracted; the next day he did not feel well about the lungs; about a week after, constantly feeling worse, he consulted a physician, who told him his lungs were congested; three months after, he died from consumption; the gas has different effects on different individuals, in some producing depressing effects; he thought the apparatus and material examined by him were capable of producing gas sufficiently pure, providing it is used in three hours after being made.

"A Mr. Hoyt, dentist, testified that he had tried nitrous oxide gas, but that he asphyxiated or strangled about one person in fifty with it. His experience was limited. He did not understand it; he always required the presence of a physician.

VERDICT.

"We find that Mrs. Ann O. Shaughessy came to her death from asphyxia or apnœa, as evidenced by the symptoms manifested by the patient before death and the conditions found at the post-mortem, the asphyxia having, in our opinion, been induced by the inhalation of gas administered.

"The jury further agree in the opinion, that any other than the prescribed method of preparation of nitrous oxide gas as to temperature for generation and appropriate washing bottles, is to be regarded as a careless method of manufacture. In this particular we regard the apparatus used by Mr. Newbrough as imperfect.

"Furthermore, we regard the administration of nitrous oxide gas as reckless where each administration is an experiment upon the patient, which it must be where there is not a positive certainty as to the chemical purity of the gas, and ignorance in the administration of the physical condition of the patient, and the physiological effects manifested by anæsthesia.

"Furthermore, we regard it as necessary for safety that an administrator of nitrous oxide gas should have a knowledge of the application of restorative agents, adapted to alarming conditions in anæsthesia, and be always supplied with such agents."

(Signed) Stephen Rogers, M. D.; Faneuil D. Weisse, M. D.; Richard J. O'Sullivan, M. D.; Albertus L. Vandewater, M. D.; Augustus Wahlfarst, M. D.; Edward Frankel, M. D.; Franz Henel, M. D.; John T. Nagle, M. D.; and Frederick W. Lillienthal, M. D.

By the old process the gas is passed through three or four bottles of water, of one gallon each, and in at the top of the reservoir of water. By my process it passes only through one gallon wash-bottle, but it is finely divided in the strainer and washed in a tank of one hundred and twenty gallons.

The lamp is also regulated to produce the correct amount of heat. In justice to the medical jury who pronounced my apparatus as imperfect, I would say they were undoubtedly misled by the chemist Endermann. This witness never examined my apparatus. He merely went into the laboratory, looked at the gasometer a moment, and then walked out, asking for a little of the water in the wash-bottle. When we returned into my office, I suggested he had better go back in my laboratory and examine the whole apparatus, and he said, "Oh! no; dat make no difference. We make dat all right." I then gave him some nitrate of ammonia, as he said to analyze the gas; but because the gas had just been made he deferred it till next morning. The third time he came, I asked him for the analysis of the nitrate of ammonia. It was Powers & Weightman's make. He said, "Oh! dat's all right!" I procured some gas in a bag for him to analyze, but he did nothing of the sort; he merely put a piece of paper, moistened with some solution, into the mouth-piece, and turned the gas on it for a little while. Said I, "Do you call that the way to analyze gas? I do it in an entirely different way." He replied, "Oh! dat's all right! Dat gas is pure enough." And he and his friend departed.

In his testimony he said he made gas with my apparatus. That is false. He made no attempt whatever to do so. He also testified that after the death of Mrs. O'Shaughnessy, I let the gas all escape, so he could not test it. That is also a downright falsehood. That lot of gas was not let off at all, but used up in the regular way during the next day. Dr. Colton called the morning after the accident to take of the same gas and did so. He pronounced it excellent. The other part of the testimony of Enderman, about his friend dying from the effects of nitrous oxide gas, is most probably on a par with his statement of my gas apparatus.

Statement of Thomas Clear:

I was in the laboratory of Dr. Newbrough when the chemist Endermann called. He did not make an examination of the apparatus for making nitrous oxide gas. His testimony that the gas of which Mrs. O'Shaughnessy inhaled was allowed to escape, is false. Mr. Endermann never made gas in Newbrough's apparatus. I work at that myself.

Statement of John Griffiths:

I was present when Mr. Endermann visited the laboratory of Dr. Newbrough. Endermann made no examination of the apparatus for making nitrous oxide gas. His testimony that the gas, of which Mrs. O'Shaughnessy inhaled, was allowed to escape, is false. It is also false that he made gas with Newbrough's apparatus.

Statement of G. Q. Colton:

There are three facts with this death to be noted:

*First.* The lady had her teeth extracted while *not* under the influence, and during the operation fainted.

*Second.* The nervous system received more or less of a shock from the pain of extraction.

*Third.* The lungs were found in a state of asphyxia.

There are three answers to these facts:

*First.* Had the lady properly inhaled the gas, *she could not have fainted*, because the gas, instead of arresting, increases the circulation.

*Second.* She would have experienced no pain, and, of course, no shock to the system.

*Third.* There would have been no asphyxia, as the gas, containing as it does more oxygen than the air, rapidly oxygenizes the blood, and *prevents* asphyxia or congestion. The ablest medical authorities on nitrous oxide recommend that when asphyxia is threatened from any cause, the gas should be administered as the quickest and most effectual remedy. Indeed, one of the most distinguished professors of chemistry in our city maintains that a law should be passed compelling dentists and surgeons, when administering *chloroform*, to have some nitrous oxide gas on hand, to restore the patient in case asphyxia or congestion is threatened.

"I do not mean to intimate that caution and care should not be exercised to make pure gas, and to administer it properly; but there was no evidence presented in this case to show that Dr. Newbrough was at fault in these respects.

"In view of these facts, I can not conceive how the jury could form an 'opinion' that this 'death from asphyxia' was 'induced by the inhalation of gas administered.' Now, in point of fact, the amount of gas previously inhaled, according to all the testimony on the subject, was not sufficient to produce any appreciable effect on the system; consequently there was no *evidence* to justify the 'opinion' arrived at by the jury. Not a single witness, not even the medical gentlemen who were examined, expressed any such opinion.

"It is a little singular that none of the distinguished professors of chemistry in our colleges—such as Professor Draper, Professor Doremus, Professor Chandler, etc., men known to be experts in the manufacture of nitrous oxide, and of its effects on the human system, were called in to give testimony. These men were not called, but in their stead a number of dentists and physicians whose experience with the gas has been very limited, and who could not tell the constituent elements of the salt from which it is made. There was one chemist called, who stated that he had seen but *six persons* under the anæsthetic influence of the gas!"

As the medical jury were at a loss how to examine witnesses in this case, it is suggested that hereafter the following questions, or something of the same nature, be handed to any similar body should occasion ever require it.

What is nitrous oxide gas? What is its effect on a healthy person? On a person affected with old pleuritic adhesions? In pulmonary apoplexy? In what manner does the respiration behave under pulmonary tuberculosis? In acute phthisis? Does the breathing of nitrous oxide gas denote the presence of any of these diseases, and how? Does it make the oscultation plainer? By what manner of respiration can you determine the indaptability of nitrous oxide to produce a healthy insensibility, and *vice versa?* How many cases, where physicians did diagnose lung diseases, have you seen brought under the influence of nitrous oxide gas? And the result? Can you give us a list? What is the behavior of a patient afflicted with fatty degeneration of the heart, while under the influence of nitrous oxide? How do you know it? After your experiments on animals, in what condition did you find the heart? The same with nitrous oxide? How with sulphuric ether? Is there a certain condition in which you always find the heart when death has been caused by nitrous oxide gas? Is there even a probability that death was caused by nitrous oxide gas when the cavities of the heart are found empty? What is the smallest quantity of chloroform that will produce an appreciable effect on the system? If suddenly applied? If largely diluted with air? The same of nitrous oxide? Of ether? What is the longest period of time you have kept an animal unconscious to pain, and yet resuscitated him?

There are few experts in giving nitrous oxide gas but wished the recent examination would have taken this course. A number of dentists were ready for such examination; but the subject was not gone into, though the verdict is given as if it had been so.

---

